# Enhanced gastric cancer classification and quantification interpretable framework using digital histopathology images

**DOI:** 10.1038/s41598-024-73823-9

**Published:** 2024-09-28

**Authors:** Muhammad Zubair, Muhammad Owais, Tahir Mahmood, Saeed Iqbal, Syed Muhammad Usman, Irfan Hussain

**Affiliations:** 1 Faculty of Information Technology & Computer Science, University of Central Punjab, Lahore, Punjab Pakistan; 2 Khalifa University Center for Autonomous Robotic Systems (KUCARS) and Department of Mechanical & Nuclear Engineering, Khalifa University, Abu Dhabi, United Arab Emirates; 3 Division of Electronics and Electrical Engineering, Dongguk University, Seoul, Korea; 4 Department of Computer Science, School of Engineering and Applied Sciences, Bahria University, Islamabad, Pakistan

**Keywords:** Cell biology, Cancer, Biomedical engineering

## Abstract

Recent developments have highlighted the critical role that computer-aided diagnosis (CAD) systems play in analyzing whole-slide digital histopathology images for detecting gastric cancer (GC). We present a novel framework for gastric histology classification and segmentation (GHCS) that offers modest yet meaningful improvements over existing CAD models for GC classification and segmentation. Our methodology achieves marginal improvements over conventional deep learning (DL) and machine learning (ML) models by adaptively focusing on pertinent characteristics of images. This contributes significantly to our study, highlighting that the proposed model, which performs well on normalized images, is robust in certain respects, particularly in handling variability and generalizing to different datasets. We anticipate that this robustness will lead to better results across various datasets. An expectation-maximizing Naïve Bayes classifier that uses an updated Gaussian Mixture Model is at the heart of the suggested GHCS framework. The effectiveness of our classifier is demonstrated by experimental validation on two publicly available datasets, which produced exceptional classification accuracies of 98.87% and 97.28% on validation sets and 98.47% and 97.31% on test sets. Our framework shows a slight but consistent improvement over previously existing techniques in gastric histopathology image classification tasks, as demonstrated by comparative analysis. This may be attributed to its ability to capture critical features of gastric histopathology images better. Furthermore, using an improved Fuzzy c-means method, our study produces good results in GC histopathology picture segmentation, outperforming state-of-the-art segmentation models with a Dice coefficient of 65.21% and a Jaccard index of 60.24%. The model’s interpretability is complemented by Grad-CAM visualizations, which help understand the decision-making process and increase the model’s trustworthiness for end-users, especially clinicians.

## Introduction

Gastric cancer (GC), recognized as one of the predominant malignancies, stands among the top five most frequent types of cancer worldwide, as outlined by the World Health Organization (WHO) report^1^. The startling data shows that 7% of all cancer cases and an incredible 9% of cancer-related deaths are attributable to patients with GC. Patients with this disease have an inferior prognosis, with a disease-specific survival rate of about 12 months. Sadly, 90% of cases of GC end in death within the first five years of the diagnosis. Medical professionals’ accurate prognostic estimation of GC is of utmost importance due to its aggressive and frequently fatal nature^2^. This emphasizes the importance of pursuing thorough research to improve patient outcomes by expanding the available knowledge of GC, enabling early detection, and raising prognostic accuracy.

In recent years, the use of image analysis systems has increased significantly, especially in intelligent diagnosis. Notably, several deep learning (DL) techniques have been created and implemented for gastric histopathology classification and segmentation (GHCS), with convolutional neural networks (CNNs) at the forefront. The evolutionary path of DL algorithms from VGG-16 to fully convolutional networks (FCNs) and later to DeepLab in GHCS highlights the ongoing progress in utilizing state-of-the-art (SOTA) technologies for GC analysis. GC is diagnosed primarily through endoscopic examinations, biopsies, and histopathological analyses^3^. Although hematoxylin and eosin (H&E)-stained pathological biopsies are currently the gold standard for diagnosis and offer valuable information about tumor characteristics like histological type, grade, and stage, there are still inherent limitations. These techniques’ inherent challenges with potential sampling mistakes and invasiveness could all negatively impact the accuracy of the diagnosis^4^. Even with careful examination of tissue under the microscope, pathologists’ expertise and involvement in diagnosis could lead to differences in the interpretation of tissue pathology images. Furthermore, due to the intense workload that pathology professionals face and the subjectivity associated with human understanding, errors may occur in GC detection^5^.

Advancements in medical technology have led to the development and implementation of Computer-Aided Diagnosis (CAD) systems, revolutionizing the screening and diagnostic processes across various medical experts. Developing a CAD system becomes essential to addressing the above-mentioned issues. Since CAD systems are already effectively used to diagnose conditions affecting the skin^6–8^, retina^9–15^, breast^16,17^, lungs^18,19^, brain^20–22^ and many other organs, their introduction marks a significant advancement in improving accessibility, efficiency, and accuracy in healthcare. CAD systems enhance medical professionals’ diagnostic abilities by utilizing advanced algorithms and machine learning (ML) techniques, which allow them to interpret medical images more quickly and accurately. The CAD system can also detect abnormal regions in pathological GC images and segment them proficiently. Doing this seeks to lessen the likelihood of incorrect diagnoses during histological examinations and address the shortage of pathologists^23^. The integration of sophisticated algorithms into this CAD system has the potential to improve early diagnosis significantly.

In most studies, endoscopy^24–28^ is predominantly used for GC segmentation because it provides a quicker and less labor-intensive method for visual examination. However, whole-slide digital histopathology images (WSDHI) offer more detailed and precise information but require time-consuming and tedious manual analysis. Histopathology images enable microscopic examination^29^, which is necessary for the accurate diagnosis and staging of GC. While endoscopy offers a macroscopic view, these images allow for identifying subtle cellular abnormalities and variations in tissue structure. Understanding the size and makeup of cancerous areas is crucial for clinicians’ decision-making.

Automatic GC segmentation is a critical task in digital pathology image analysis. The challenges in this task are due to the non-rigid characteristics of cancerous regions, indefinite boundaries, and varying sizes. These complexities have proven difficult for existing approaches, requiring sophisticated methods. One of the studies on GC segmentation has explored the DL-based method by integrating customized modules like deformable and Atrous convolutions, Atrous Spatial Pyramid Pooling, and encoder-decoder-based networks for multi-scale segmentation^30^. Another study proposed an overlapped region forecast algorithm, and a novel neural network architecture is presented in a different study for automatic GC segmentation. It presents a framework for reiterative learning with minimal manual annotation. In addition, the overlapped region forecast algorithm addresses patch boundary errors, and the study modifies the loss function to improve model convergence and prevent local minima. By incorporating predictions and iteratively training with sparse annotations, the study considerably raises the quality of training data^31^.

Although fully supervised models have produced impressive tissue segmentation results, they demand a high processing overhead and annotations at the pixel level. To overcome this, a recent study achieved tissue segmentation using only patch-level classification labels through weakly-supervised methods. This method uses a two-step model: Multi-Layer Pseudo-Supervision fills in the gaps between pixel-level and patch-level annotations after a CAM-based model creates pseudo masks from patch-level labels^32^. When it comes to the field of intelligent GC diagnosis CNNs, current approaches typically focus on specific features or network topologies without a plan for gathering spatially comprehensive data. By characterizing spatial relations, the conditional Field, which is well-known for its efficacy in analyzing complex images, provides a solution. An innovative Hierarchical Conditional Random Field technique was used in a study to identify aberrant areas in digital histopathology pictures automatically. To improve segmentation accuracy, the model combines patch-level and pixel-level data with higher-order potentials and uses graph-based post-processing. To accomplish this, three additional CNNs are tuned to generate patch-level potentials, guaranteeing strong spatial segmentation, while a CNN is trained for pixel-level potentials^33^.

DL employs two primary techniques in lesion identification: detection and segmentation^34–36^. The detection method involves region-level classification^37^, while segmentation entails pixel-level classification^11,38,39^. Compared to detection, segmentation offers more intricate insights. In clinical settings, pixel-level classification enhances diagnostic accuracy by allowing precise sizing of lesions. Furthermore, it facilitates tracking changes in lesion size and shape over time, utilizing shape as a reference point during detection. This approach broadens the scope of evaluation beyond mere diameter measurements, incorporating lesion area into treatment efficacy assessments. The proposed methodology takes the concept of CAD to a new level by utilizing effective algorithms designed to perform remarkably well in segmentation and classification tasks. While most existing CAD systems focus primarily on classification, our system goes one step further and achieves superior anomalous area segmentation. Accurately quantifying the spread of abnormalities is necessary to estimate the stage of GC, and highly precise segmentation methodology makes this possible. By giving precise information about the degree and kind of abnormalities, the suggested system enhances the diagnosis process and makes GC staging more accurate. In short, the limitations of the current diagnostic techniques, such as manual interpretation, correct quantification, speedy screening and diagnosis, and the lack of pathologists, lead to the requirement for an enhanced CAD system to classify and segment GC. The proposed system overcomes these challenges with an advanced GHCS that effectively performs cancerous spread quantification and classification. The following are this research study’s primary contributions:

- Proposed an automated and efficient GC classification and segmentation framework using WSDHI.
- Introduced an enhanced version of the Naïve Bayes (NB) classifier leveraging the Gaussian Mixture Model (GMM) for improved classification accuracy.
- Utilized Expectation Maximization (EM) to refine the Gaussian model, further enhancing its performance in GC classification.
- Evaluated the classifier on two publicly available datasets, demonstrating promising results that underscore the versatility and effectiveness of the proposed approach.
- Developed a novel improved Fuzzy c-means algorithm for segmentation, enabling accurate quantification of abnormal regions in GC histopathology images.
- Demonstrated superior performance of the proposed classification and segmentation module compared to existing competitive methodologies, highlighting its potential for advancing GC diagnosis and treatment.
- The Gradient-weighted Class Activation Mapping (Grad-CAM) visualizations provide the model’s interpretability by highlighting the abnormal regions in histopathology images used for classification and segmentation purpose.

## Materials and methods

**Fig. 1:**
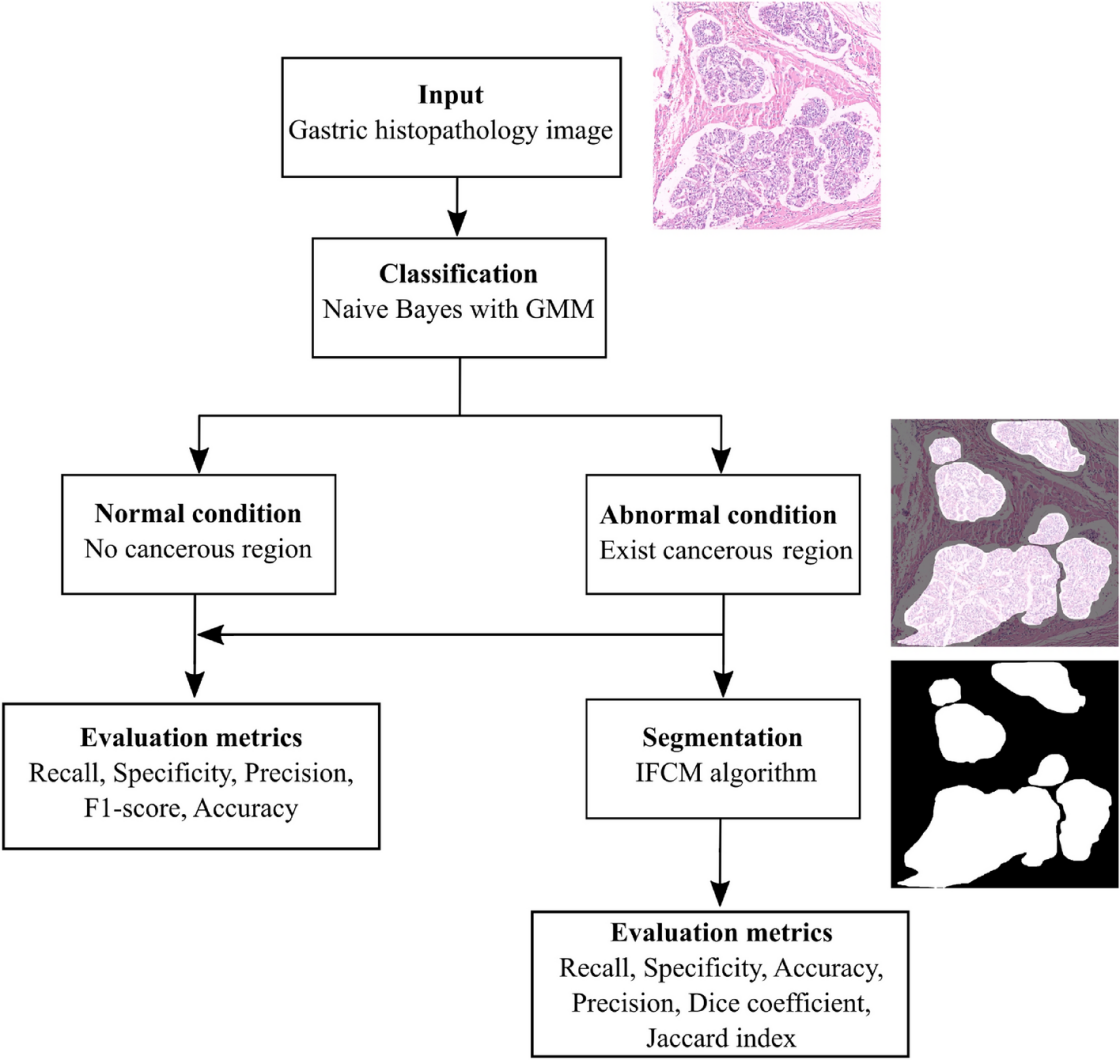
Flow chart of the proposed framework for gastric histopathology image classification and segmentation.

This section comprehensively explains the proposed framework, including data preprocessing, classification, and segmentation of the region of interest. The flow chart of the proposed framework is illustrated in Figure 1.

### Experimental Settings

#### Dataset

In this study, we have used two publicly available datasets for classification to show the proposed classifiers’ versatility better. However, one dataset is used for segmentation purposes due to non-available annotations for the second dataset. The details of these datasets are as follows. There are 245,196 images in the publicly available histopathology image dataset GasHisSDB^40^. This dataset comprises 97,076 abnormal images and 148,120 normal images and is divided into three subsized image datasets. The complete dataset is split into training and testing sets, keeping a 70:30 distribution to make training and testing sets. In addition, the training data is further divided, with a 70:30 random distribution of training and validation sets. Table 1 contains comprehensive distribution data.

**Table 1:** Information of data splitting for training, validation, and testing for GasHisSDB^40^ dataset.

**Image type**	**Training**	**Validation**	**Testing**
Normal	75264	25499	47362
Abnormal	44882	25997	26197
Sum	120146	51491	73559

Another dataset for gastric histopathological images stained with H&E is used, which is publicly available^41^. The dataset consists of 140 normal images without ground truth (GT) images and 560 abnormal images with respective GT images labeling the positive regions or cancerous cells. Positive regions appear in abnormal images, while no positive regions appear in normal images. All the gastric histopathological images have a $2048 \times 2048$ pixels resolution. The image formats in the dataset are “tiff” and “png”. The abnormal regions in the histopathology images are labeled by very skilled pathologists.

#### Data augmentation

The dataset^41^ consisted of 700 images, which might not be enough for an ML model to be trained, especially in applications related to medicine where a larger dataset is typically preferred. Data augmentation techniques are employed to expand the dataset to get around this restriction. Image transformations, including flipping the image horizontally and vertically and rotating it in 45, 90, 135, 180, 225, 270, and 315 degrees. These transformations increase the dataset size, yielding ten augmented versions of each original and GT image.

It is to be noted that the data augmentation process does not enhance our classification methodology, as the manually selected feature set does not benefit from the typical transformations applied during augmentation. The augmentation is necessary for a fair comparison with the performance of other classifiers. Data augmentation is important to ensure a rigorous and fair comparison of other model’s results compared to our proposed model. The comparative DL models, unlike our approach, rely heavily on large and diverse datasets to reach their full potential. Augmentation was therefore employed to increase the HCRF dataset, enhancing the training, validation, and testing samples available to these models. This step was necessary to provide them with the data diversity required to perform optimally and to allow for a meaningful comparison of results. The original gastric histopathology images are cropped into more manageable, smaller 256 by 256-pixel images to address their size. This helps to overcome computational challenges associated with processing large images. By cropping the original images, the augmentation increases the size of the dataset. In particular, the original dataset’s total size is multiplied by ten due to rotational and flipping augmentation and then further to 64-folds, as every original image is cropped to a uniform 256 by 256-pixel size. For example, 64 cropped images are produced when a single input image with a resolution of 2048 by 2048 pixels is cropped. The normal and abnormal classes are split into a ratio of 60:20:20 and are used for training, validation, and testing purposes. The data augmentation and splitting information can be seen in Table 2.

**Table 2:** Dataset^41^ splitting for training, validation, and testing before and after data augmentation is applied.

**Image type**	**Training**	**Validation**	**Testing**
Original Normal	84	28	28
Augmented Normal	53760	17920	17920
Original Abnormal	336	112	112
Augmented Abnormal	215040	71680	71680

**Fig. 2:**
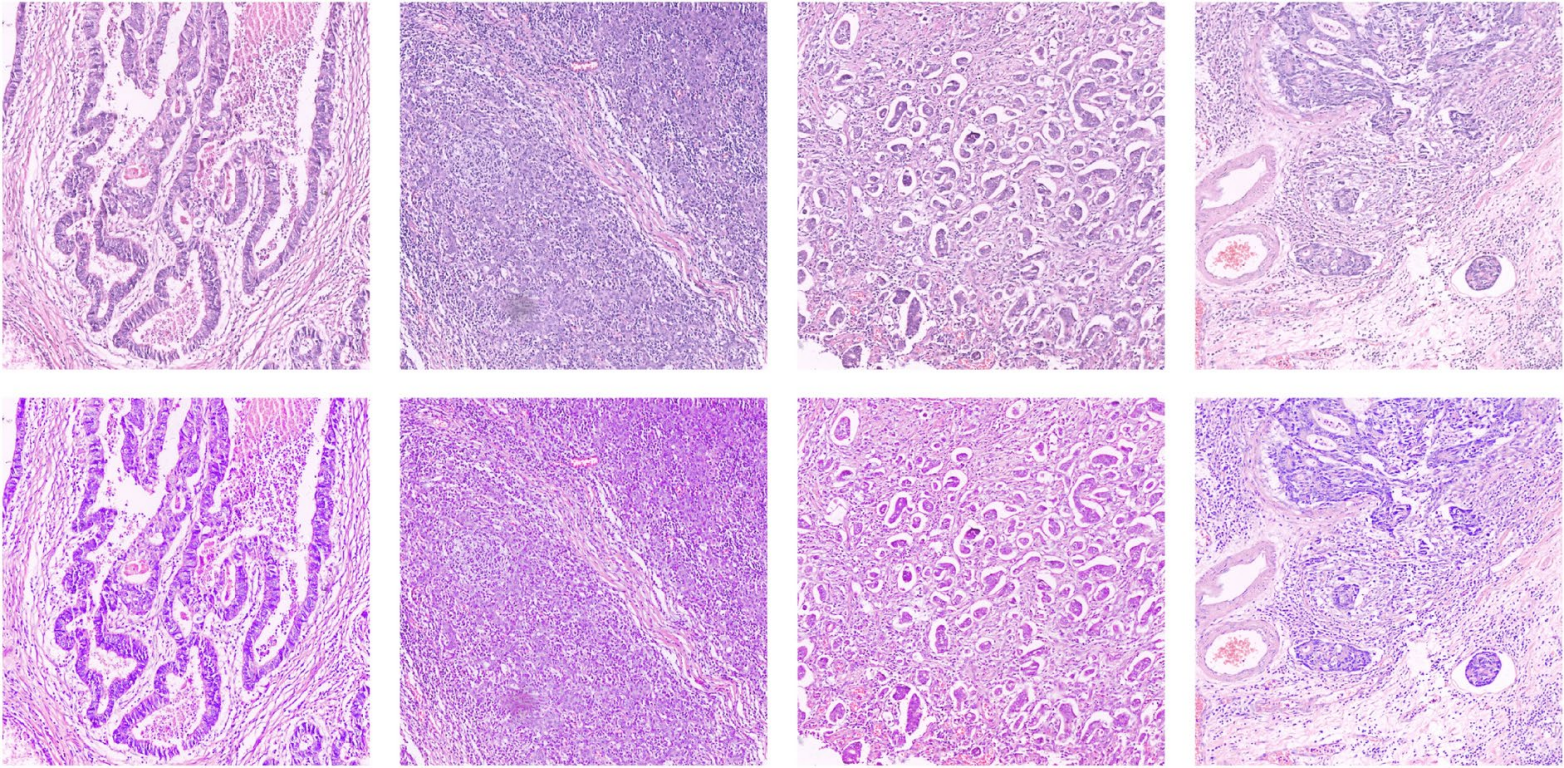
Examples of applied normalization results. The images in the upper row show the original dataset images without normalization, whereas the lower row illustrates the images obtained after applying the normalization.

Histopathology images are considered gold standards for cancer diagnosis; however, the staining process can introduce variability because of different staining materials, inconsistent techniques, and differences in scanners. This color variation could negatively affect the result of a CAD system. Stain normalization is helpful by standardizing images, reducing imbalances, and enhancing visual clarity. This increases the precision of ML/DL models by emphasizing tissue architectures rather than color variations. The robustness and overall performance of automated diagnostic methods are strengthened by color normalization, which is essential for the dependability of CAD systems. We applied the technique proposed by Reinhard^42^ for staining normalization, which transfers color between a reference image and a color-varied image using the mean and variance. A few results of the normalization process on the applied images are illustrated in Figure 2. In this normalization method, the source image is adjusted to match the target image by aligning its color distribution through a linear transformation in the perceptual color space. This process ensures visual consistency across images, aiding in accurate analysis.

### Classification of gastric histopathological images

To determine the optimal course of therapy, abnormalities must be precisely identified and the condition’s progression monitored. Positive outcomes have been shown for automated abnormal region identification using ML techniques, including traditional and DL classifier-based approaches. These classifiers use features from the H&E images to distinguish between normal and abnormal structures. The foundation for a future discussion of the various classifiers used to classify GC is laid out in this paragraph. It highlights the importance of accurate and efficient methods for improving the histological image-based diagnosis of GC.

Classification has traditionally been accomplished using conventional or neural network-based classifiers. If the feature space lacks linearity in separation, these classifiers are unsuitable. Classification has traditionally been accomplished using conventional classifiers or neural networks. While conventional classifiers struggle with non-linear separability in the feature space, neural networks are designed to handle such complexities, making them suitable for non-linear classification tasks. The NB classifier has some distinct advantages over the traditional classifiers for segmenting anomalies. Because of its computational efficiency, it is appropriate for large datasets. It can handle high-dimensional feature spaces effectively. Additionally, NB lowers model complexity under the assumption of feature independence. NB is a viable solution for Gastric Histopathology Classification (GHC) tasks because of its rapidity and precision. All potential abnormal regions are extracted during the candidate abnormal region phase segmentation. A feature set is defined for the classification task to identify cancerous regions from the WSDHI. Vector V, comprising all the specified features for image I, is established as V = {f1, f2, f3, . . . . fn}.

Previous studies have used various visual features from WSDHI as prerequisites for ML-based classification. Commonly used features such as Histogram of Oriented Gradients (HOG), Color Histogram (CH), Luminance Histogram (LH), Local Binary Patterns (LBP), and Gray-Level Co-occurrence Matrix (GLCM) have established a solid foundation for image analysis, proving essential in various classification tasks^43^. However, in our study, we opted for multiple manually selected features due to their ability to capture critical aspects of gastric histopathology images. The multiple manual features are relevant to medical imaging and contain structural information that overall improves the classification task for histopathology images. The salient features can effectively define the ROI due to the enhanced discriminative power, resulting in better classification. Hence, multiple feature selection is critical in characterizing the gastric histopathology images. Table 4 lists and briefly describes the chosen features and their aspect-based category.

The manually selected features are designed to capture specific characteristics of gastric histopathology images, complementing the strengths of ML methods. Meanwhile, DL has shown superior performance, especially with properly normalized data. Our approach highlights the potential benefits of combining handcrafted features with ML techniques in certain contexts. In a comparative analysis, the manually chosen features have demonstrated superior performance in the WSDHI classification task. A significant improvement in the classification task is shown in Table 3, where the evaluation performance comparison of the individual commonly used features and the multiple manually selected features is shown. It is to be noted that all five individual features (CH, LH, HOG, LBP, GLCM) were tested using six different classifiers (LR, KNN, RF, Linear SVM, Non-linear SVM, ANN). However, in table 3, we have only shown the results of classifiers that give the best accuracy for the individual features on images from 160 x 160 pixels sub-dataset of GasHisSDB^43^.

**Table 3:** Classification performance comparison based on single feature versus multiple manually selected features. [In (%)].

**Features**	**Classifier**	**Class**	**Rec.**	**Spe.**	**Pre.**	**F1**	**Acc.**
Color histogram^43^	Random Forest	Normal	84.55	87.83	89.88	87.13	
		Abnormal	87.83	84.55	81.65	84.63	85.99
Luminance histogram^43^	Random Forest	Normal	80.78	76.60	84.14	82.42	
		Abnormal	76.60	80.78	72.17	74.32	79.13
HOG^43^	ANN	Normal	91.57	15.40	62.45	74.26	
		Abnormal	15.40	91.57	54.30	23.99	61.54
LBP^43^	LR	Normal	82.02	62.42	77.03	79.45	
		Abnormal	62.42	82.02	69.32	65.69	74.29
GLCM^43^	LR	Normal	75.00	65.85	77.14	76.06	
		Abnormal	65.85	75.00	63.16	64.48	71.39
Feature set from Table. 4	**Proposed** **NB with GMM**	Normal	98.98	97.76	98.88	98.90	
		Abnormal	97.76	98.98	98.24	97.93	98.52

The proposed approach employs a Bayesian classifier based on the Gaussian function, bypassing traditional decision boundaries, which produce encouraging results. As a result, the suggested methodology achieves a high level of accuracy. The GHC threshold value is intentionally set low to guarantee that all abnormal areas are recognized during the classification process. A supervised classifier that uses datasets for testing, validation, and training has been used. We have employed a labeled dataset for training, and the outcome is determined by the Bayes decision rule, as expressed in equation 1.

1 $$\begin{aligned} F(K|C_2) > F(K|C_2)F(C_1) \end{aligned}$$

Equation 2 is used to compute the $F(K|C_l)$, representing the class’ probability density function (pdf). The pdf enables probabilistic decision-making, which is important in classification tasks. This would improve model accuracy overall, handle uncertainty in the data, and help identify relevant features. The PDF is the mathematical backbone of many probabilistic classifiers, including Gaussian Naïve Bayes, allowing them to make informed predictions based on the distribution of features within each class. The ratio of class $C_l$ samples in the training set and the prior probability of class $C_l$ are represented by the $F(C_l)$. For any class, equation 3 calculates the feature vector’s probability density function using multivariate Gaussian probability density function.

2 $$\begin{aligned} {F(K|C_l) = \sum \limits _{i=1}^{s_l} G\Bigl (w|\mu _{i},\sum i\Bigl )h_i} \end{aligned}$$

$F(K|C_l)$ is the x-dimensional Gaussian distribution with weight $h_i$. Meanwhile, the count of Gaussian mixtures for classification is represented by $s_l$. The classes for classification are represented as $C_i = (C_1, C_2)$. The count of Gaussian mixtures in classification using GMMs refers to the number of Gaussian components used to model the distribution of each class. This count is significant because it establishes how flexible the model is in capturing the distribution of the underlying data and because it directly affects the computational efficiency and performance of the classifier.

3 $$\begin{aligned} L\Bigl (w \; \mu , \; \sum \Bigl )=\frac{1}{(2\pi )^\frac{q}{2} |\sum |^2} \exp \Bigl (\frac{-1}{2} (w-\mu ) \sum ^{-2} (w-\mu )\Bigl ) \end{aligned}$$

Where the product of feature vector *w* and mean vector $\mu$ yields the number of features, *q*. The feature vector $\mu$ contains the mean of every feature. The covariance matrix $\sum$ has dimension $q \times q$.

The model offers rapid testing at the cost of a longer training period. Non-parametric techniques do not constrain the underlying data but are challenging to compute. However, it guarantees a speedy testing phase, independent of the quantity of training samples and solely contingent upon the selection of *s*. The ideal value of *j* to maximize the GMM accuracy is calculated by applying EM to multiple validation sets randomly selected from categorized training data. EM enhances parameter estimation by iteratively optimizing the likelihood function, allowing the model to capture complex data distributions accurately. This refinement improves separation between classes, resulting in higher classification accuracy. Additionally, EM’s ability to converge to local optima ensures that the model parameters are optimally adjusted, making the approach both robust and effective in handling diverse data. By determining the local maximum pdf of the training data set, the EM finds the optimal value for *s* by an iterative technique. The EM sets the value of *s* at which s-weighted Gaussians can accurately represent the data. Our suggested approach combines EM with supervised learning, in which we use prior knowledge to guide the training procedure. When estimating parameters, supervised methods yield higher accuracy than unsupervised methods, which rely on a large amount of data to be forecast. In the EM learning process for GMM, the variables for different values of *s* and weights *h* are trained. This process consists of two stages: estimation and maximization. The first step is the probability that a Gaussian process is produced. In the second step, parameter adjustment increases the data’s likelihood.

The rationale behind combining EM with supervised learning in our approach is rooted in the strengths as supervised learning leverages prior knowledge by using labeled data to guide the training process, which significantly enhances the accuracy of parameter estimation. Meanwhile, EM integration refines the GMM by iteratively optimizing parameters through the EM process. The two-stage EM process estimation and maximization-allows for more precise model tuning. The probability that a data point belongs to a particular Gaussian component is determined in the estimation step, and the parameters are changed in the maximization step to raise the likelihood that the data will fit the current model. Our model performs better on GC classification because of the combination of supervised learning’s guided training and EM’s iterative refinement, which guarantees higher accuracy and robustness in parameter estimation.

**Table 4:** List of manually selected aspect-based features with a short description.

**Feature’s aspect-based category**	**Feature**	**Short description**
Relevance to medical image	Mean value	The mean brightness of each pixel in the original image
	Mean saturation	The mean pixel’s value from the color channel
	Compactness	$$p^2/4$$$$\times$$$$\pi$$$$\times$$*A*, where p = perimeter and A is the total area
	Mean intensity	The mean of all the pixel intensity values within the candidate region in a green channel
	Area	Sum of all pixels in a specific area
	Mean hue	It depicts the spectral color in its purest form, devoid of any black or white mixture
Structural information	Total energy	The total number of pixels in the candidate region divided by their total intensity sum.
	Total entropy	All pixels’ value in a square area
	Mean gradient amplitude	The mean of all the highlighted pixels with the ability to distinguish between distinct and fuzzy contours
	Third-moment value	Measure of skewness of the candidate region.

### Segmentation of gastric histopathological images

Cancerous areas are visually recognised in H&E stained slide images by examining cellular and tissue properties. Pathologists search for anomalies in cell nuclei, such as nuclear pleomorphism, hyperchromatism, and irregularities in size and shape. Critical indicators of cancerous regions include hypercellularity, irregular cell borders, stromal changes, tissue necrosis, increased mitotic activity, and subtle colour variations. Furthermore, ML and image analysis techniques can be used to automate the identification process by identifying patterns associated with malignant tissues. Computational methods aided in this visual analysis are essential for an accurate cancer diagnosis and prognosis.

The standard Fuzzy c-means clustering algorithm has robust characteristics for ambiguity and retains more information than complex segmentation techniques. However, because it is an iterative algorithm, its main drawback is that it is computationally greedy. We recently developed an Improved Fuzzy C-Means (IFCM) algorithm. In the IFCM, cluster updates and membership value criteria are the only requirements. Consequently, the advantages of FCM are retained while the excessive time commitment is addressed. Equation 4 mentions the IFCM function $P_{min}$.

4 $$\begin{aligned} { P_{min} } = \sum \limits _{x=1}^{a} \sum \limits _{y=1}^{b} \biggl [ (u_{xy})^z \Big \Vert e_x - f_y\Big \Vert ^2 + G_{wi} \biggr ] \end{aligned}$$

where, E = $\lbrace$ e1, e2, e3 ... ex$\rbrace \subseteq R^{p}$, *b* is the number of clusters.

The number of data items is represented as *a*, $u_{xy}$ represents degree of membership of $e_x$ in the $y^{th}$ cluster, *z* is a weight exponent on each fuzzy membership, the prototype *z* is of the center of cluster *T*, $\big \Vert x_p - w_q \big \Vert ^2$ is the distance between object $x_p$ and cluster center $w_q$, distance between cluster center $f_y$ and object $e_x$ is measure as $\big \Vert e_x - f_y \big \Vert ^2$.

A tradeoff weighted fuzzy factor $G_{wi}$, defined in equation 5, is introduced in equation 4. The weighted fuzzy factor $G_{wi}$ balances the tradeoff between these relationships and memberships. This allows the model to incorporate both strong and weak associations between elements in a flexible, fuzzy manner. This technique is useful in classification, where capturing subtle relationships in the data is critical for accurate predictions.

5 $$\begin{aligned} { G_{wi} } = \sum \limits _{x=1}^{a} \sum \limits _{y=1}^{b} (u_{xy})^z \,\, \sum _{\begin{array}{c} x\ne y \\ y \in U_{i} \end{array}} \,\, f_{xy} \,(1-u_{xy})^z \end{aligned}$$

Where, the $u_{xy}$ is the fuzzy membership value between x and y, $f_{xy}$ is a factor associated with the relationship between x and y, *z* is the parameter that adjust the influence of $u_{xy}$, and $U_i$ is a set of indices relevant to the calculation of $G_{wi}$. Using the corresponding coordinates, for every pixel $e_x$, the similarity of the feature-weight learning is calculated. Distance of all pixels is separately calculated from the cluster’s central pixel using equation 6.

6 $$\begin{aligned} { f_{mn} } = \frac{1}{(O_{xy} +1)} \end{aligned}$$

Where, $f_{mn}$ represents the similarity measure between the pixel and the cluster center, $O_{xy}$ is the offset between the pixel $e_x$ and the central pixel in the cluster, based on the corresponding coordinates. This methodology is used in the segmentation process because it enables clustering, assembling similar pixels according to their features. The algorithm determines how strongly a pixel should be associated with a specific cluster by calculating each pixel’s similarity to the cluster center. This information then influences the learning and updating of feature weights. Algorithm 1 provides a summary of the IFCM algorithm’s steps.

**Algorithm 1:**
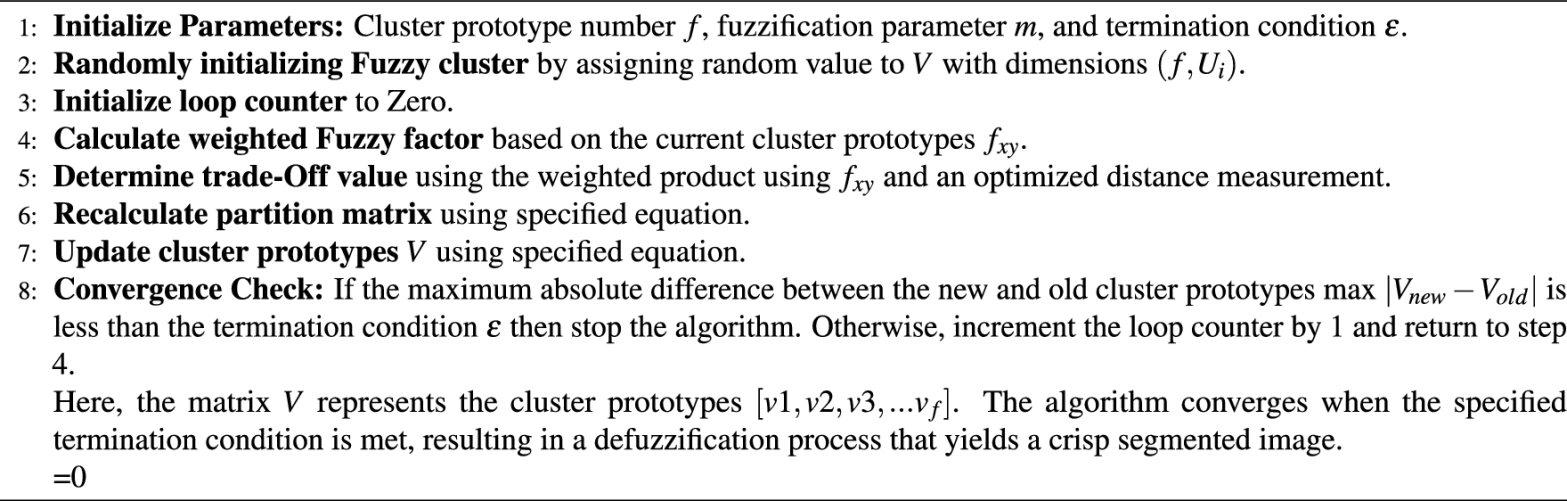
Steps of the Improved Fuzzy C-Means (IFCM) algorithm.

The IFCM algorithm significantly improves the segmentation process, especially when applied to complex dataset images, i.e., histopathology datasets. Its improved flexibility and adaptability achieved by adding a trade-off weighted fuzzy factor are among its main advantages. The algorithm can more effectively manage the variability and uncertainty present in medical data, considering the variety of patterns present in histopathology images. Additionally, the algorithm can achieve higher accuracy due to its ability to calculate weighted fuzzy factors and optimize distance measurements. This results in clusters more accurately representing the underlying data structure, leading to better-defined and more precise segmentations. Such accuracy is crucial in medical imaging, where delineating abnormal regions is essential for effective diagnosis and treatment. In addition, the algorithm demonstrates enhanced robustness to noise and oddities. Because of its fuzzy nature and weighted trade-off mechanism, it can lessen the effects of data variations and artifacts, avoiding misleading or irrelevant data points from distorting the clustering results. This robustness is especially useful in histopathology, as different kinds of noise can impact images. Furthermore, the clustering centers are continually improved to more accurately reflect the data due to the iterative update of cluster prototypes. Medical image segmentation relies heavily on more meaningful and accurate cluster centers, which are made possible by this adaptive approach. The algorithm’s convergence check ensures that the differences between the new and old cluster prototypes are more minor than the designated termination condition, resulting in a stable and dependable final clustering solution. After convergence, the defuzzification procedure yields a well-segmented image with precise and unambiguous region delineation. This is essential for real-world uses in histopathology, where the accurate and thorough segmentation of aberrant regions is required to make clinical decisions. Lastly, the enhanced FCM algorithm makes it possible to benchmark successfully against current cutting-edge techniques. The algorithm provides a valuable tool for improving the diagnostic process. It highlights its potential for progressing the field of medical image analysis by showcasing its outstanding accuracy, robustness, and adaptability capabilities.

## Results

### Evaluation metrics

The following benchmarks are employed to assess the effectiveness of the proposed approach for the classification and segmentation of GC. Among the evaluation criteria are Recall (Rec.), specificity (Spe.), accuracy (Acc.), precision (Pre.), F1-score (F1), dice coefficient (DC), and jacard index (JI). The equations to compute these metrics are listed as equation 7, 8, 9, 10, 11, 12, and 13. Moreover, confusion matrices also illustrate the performance of the classification model by providing a detailed breakdown of true positive (TP), true negative (TN), false positive (FP), and false negative (FN) predictions.

7 $$\begin{aligned} & {Rec. = TP / (TP + FN)} \end{aligned}$$

8 $$\begin{aligned} & {Spe. = TN / (TN + FP)} \end{aligned}$$

9 $$\begin{aligned} & {Acc. = TP + TN / (TP + FP + TN + FN)} \end{aligned}$$

10 $$\begin{aligned} & {Pre. = TP / (TP + FP)} \end{aligned}$$

11 $$\begin{aligned} & {F1. = 2 * Pre. * Rec. / (Pre. + Rec.)} \end{aligned}$$

12 $$\begin{aligned} & {DC. = 2 * |A \cap B| / |A| + |B| } \end{aligned}$$

13 $$\begin{aligned} & {JI. = |A \cap B| / |A \cup B| } \end{aligned}$$

### Classification of gastric cancer

In the results section, we thoroughly analyze the effectiveness of our proposed NB classifier for GC classification using H&E images. Our model demonstrated effective classification capabilities by leveraging the Gaussian function with EM. By classifying input images as either normal or abnormal, the classifier performed a binary classification and clearly indicated whether or not the presence of GC was present.

#### Qualitative results

**Fig. 3:**
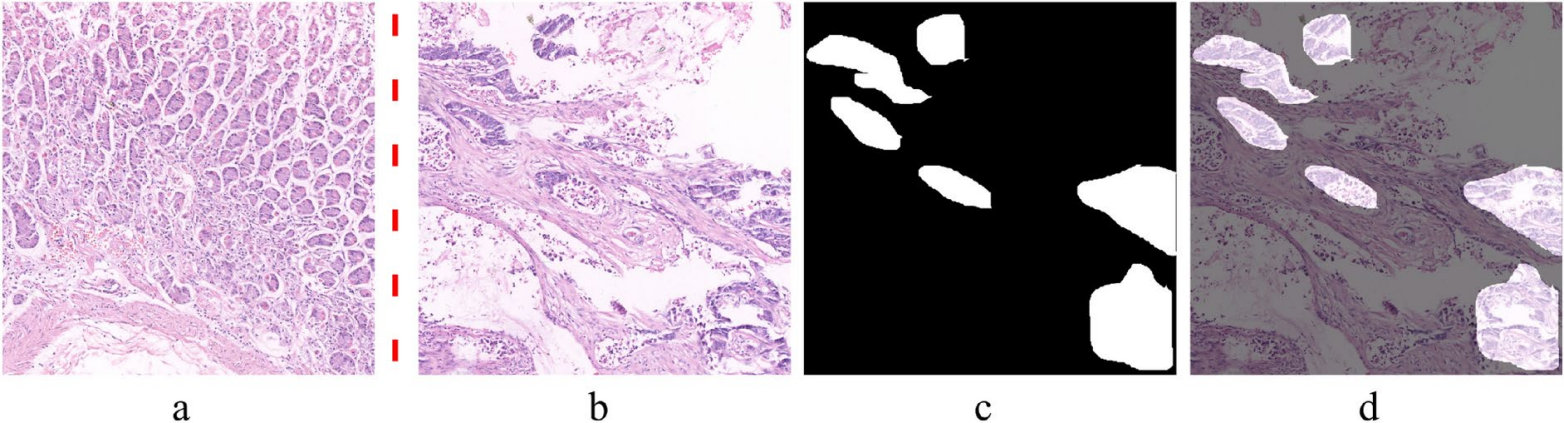
Example of classification results on HCRF dataset using the proposed classifier (**a**) Original H&E images with normal condition, which are correctly classified as normal by the proposed classifier, (**b**) Original H&E images containing abnormal regions, (**c**) Respective ground truth images provided within the dataset (**d**) Corresponding images in (**b**) with highlighted abnormal regions using the provided ground truth (**c**), which are correctly classified as abnormal by the proposed classifier.

The classifier successfully captured subtle morphological differences, such as variations in cell nuclei, tissue architecture, and overall cellular organization, associated with GC by using the defined feature set mentioned in Table 4. The results demonstrate that the classifier captured the subtle morphological differences related to GC well, such as differences in cell nuclei, tissue architecture, and overall cellular organization. In figure 3, (a) shows original images with normal conditions classified as normal. The red dotted line separates the normal from the abnormal images. (b) shows original images having abnormal regions, (c) shows the GT of the corresponding image in (b), and (d) shows the respective images in (b) with overlapped GT regions in (c), highlighting only the abnormal regions. The proposed model correctly classified the images with normal and abnormal conditions. The quantitative results are discussed in the next section.

#### Quantitative results

**Fig. 4:**
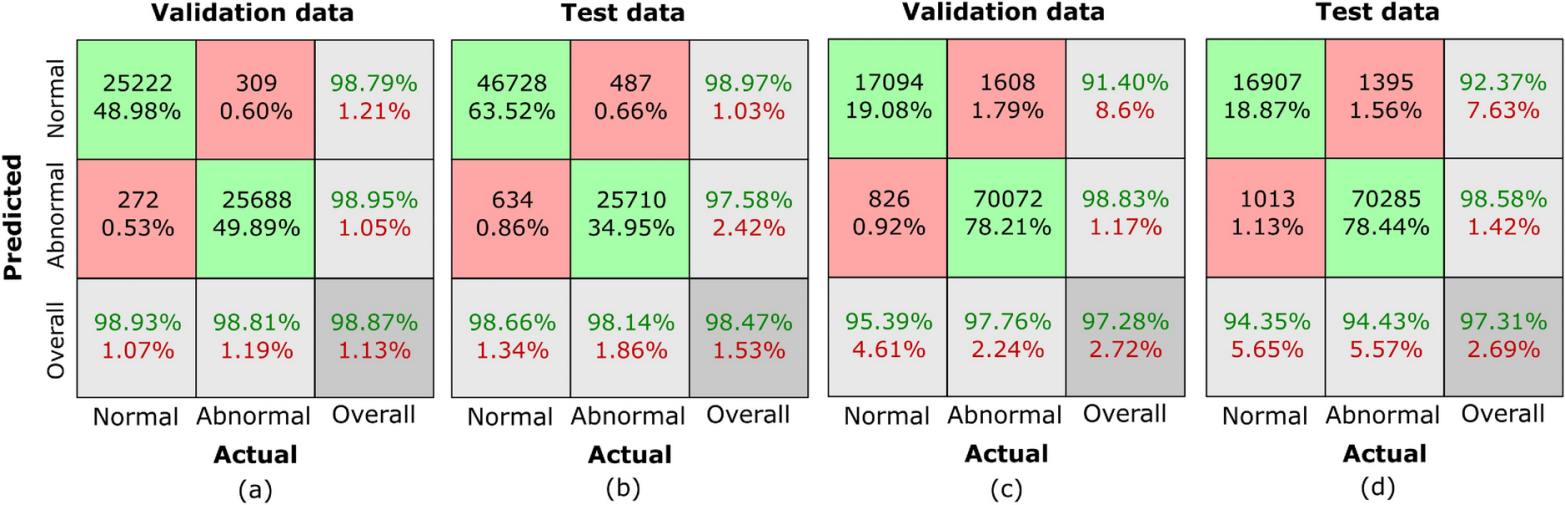
Confusion matrices **(a), (b)** GasHisSDB datasets, **(c), (d)** HCRF datasets.

We use confusion matrices to assess our proposed classifier’s performance in-depth. These matrices give a succinct representation of the degree of agreement between the model’s predictions and the real GT annotations, which is useful information about how well the model performs in classification tasks. These confusion matrices thoroughly analyse the outcomes for normal and abnormal classes. Figure 4 show classification results on GasHisSDB^40^ and HCRF^41^ datasets by our proposed classifier, respectively.

The breakdown for the normal class is given in the first column. The percentage of TN instances is shown in the first row, whereas the percentage of FP cases is shown in the second row for all input samples. The sensitivity percentage for normal cases is shown in the last row and is indicated in green. In the second column, FN and their percentage from the total sample count are shown in the first row, while TP and their percentage value from all input samples are displayed in the second row. The sensitivity percentage for abnormal cases (in green) is displayed in the last row. In the third column, the first row highlights the percentage of TN cases (in green text) from the sum of TNs and FNs, while the second row displays the percentage of FP cases (in green) from the sum of FPs and TPs. Finally, the last row provides the overall accuracy (in green). This comprehensive breakdown offers a detailed view of the performance metrics associated with each class.

The confusion matrix presented in Figure 4(a) provides a detailed breakdown of classification outcomes on the validation dataset for GasHisSDB^40^. There are 25,222 TN and 25,688 TP cases out of the total instances. Furthermore, 272 cases are classified as FP and 309 as FN. Based on the validation dataset for the standard class, 98.79% is the computed recall. The classifier’s specificity of 98.95% suggests that it can accurately identify TNs, and the derived parameters demonstrate that it can identify the TP cases with precision. Furthermore, the accuracy and F1 values (98.93% and 98.86%, respectively) were viewed as promising. Comparably, the abnormal class’s recall and specificity values are 98.95% and 98.79%, respectively, demonstrating the classifier’s outstanding ability to distinguish between abnormal and normal situations In contrast, the F1 score is 98.88%, and the claimed precision is 98.81%. The classification results for the GasHisSDB testing dataset are displayed in Figure 4(b). The 46,728 TN and 25,710 TP instances illustrate the model’s effective categorization findings. On the other hand, 487 and 634 FN and FP cases, respectively, have been documented. Recall, specificity, accuracy, and F1 score of the model in the normal class are 98.97%, 97.58%, 98.66%, and 98.81%, respectively, demonstrating its ability to accurately detect TPs and negatives in this category. The classifier’s resilience in distinguishing between anomalous and typical cases is also demonstrated by the abnormal class’s 97.58%, 98.97%, 98.14, and 97.86 recall, specificity, precision, and F1 metrics.

The confusion matrix for the validation data for the HCRF^41^ dataset is shown in Figure 4(c), revealing 17,094 TN cases and 70,072 TP cases. There are also 826 FP cases and 1,608 FN cases. The normal class’s recall, specificity, precision, and F1 metrics show values of 91.40%, 98.83%, 95.39%, and 93.35%, respectively; the abnormal class’s recall, specificity, and precision show values of 98.83%, 91.40%, 97.76%, and 98.29, respectively. Figure 4(d) portrays the results on the test dataset, with TN, TP, FN, and FP values of 16,907, 70,285, 1,395, and 1,013, respectively. For the normal class, recall, specificity, precision, and F1 score are calculated as 92.37%, 98.58%, 94.35%, and 93.34%, respectively, while for the abnormal class, recall is 98.58%, specificity is 92.37%, precision is 94.43%, and F1 score is 96.46%.

To ensure the robustness and generalizability of our proposed model, we tested our methodology on two publicly available datasets, which differ in color profiles, brightness, contrast, and other imaging attributes. Though images within each individual dataset may have undergone some standardization, they are quite different from one dataset to another. Moreover, the GasHisSDB dataset has 3 sub-datasets with different resolution images, so testing our proposed model on these two datasets shows its robustness. The performance evaluation metrics on these datasets, as presented in Table 5, demonstrate that our model consistently achieves impressive results across different conditions. By successfully validating our model on multiple datasets with inherent variability, we provide evidence that our methodology can generalize well to different imaging environments due to variability in image attributes. A complete description of the evaluation metrics is provided in Table 5.

In brief, the training and testing datasets of the GasHisSDB dataset yielded an overall accuracy of 98.87% and 98.47%, respectively. Similarly, in the HCRF dataset, the corresponding accuracies are 97.28% and 97.31%. This demonstrates that our classifier exhibits strong performance across a diverse range of histopathology images, indicating its robustness and reliability for accurate classification tasks. Promising results have been observed from automated categorization endeavors employing both traditional ML and DL^44–49^ classifier-based methodologies. These classifiers leverage diverse image features to distinguish between normal and abnormal structures. For a thorough assessment of the classifier performance, we present a detailed comparison with our proposed technique in supplementary Tables 6 and 7, shedding light on the nuanced distinctions among various classifiers.

**Table 5:** Assessing classifier’s performance on GasHisSDB^40^ and HCRF^41^ across validation and test datasets. [In (%)].

**Dataset**	**Class**	**Validation**					**Test**				
		**Rec.**	**Spe.**	**Pre.**	**F1**	**Acc.**	**Rec.**	**Spe.**	**Pre.**	**F1**	**Acc.**
GasHisSDB^40^	Normal	98.79	98.95	98.93	98.86		98.97	97.58	98.66	98.81	
	Abnormal	98.95	98.79	98.81	98.88	98.87	97.58	98.97	98.14	97.86	98.47
HCRF^41^	Normal	91.40	98.83	95.39	93.35		92.37	98.58	94.35	93.34	
	Abnormal	98.83	91.40	97.76	98.29	97.28	98.58	92.37	94.43	96.46	97.31

**Table 6:** Classification results of deep learning and machine learning classifiers on the GasHisSDB dataset. [In (%)].

**Type**	**Classifier**	**Rec.**	**Spe.**	**Pre.**	**F1**	**Acc.**
Applied DL models	Xception^44^	96.5	96.5	95.4	96.2	96.8
	Inception-V3^45^	97.3	96.1	96.2	97.2	97.1
	AlexNet^46^	91.3	90.4	89.4	92.4	91.1
	DenseNet-121^47^	91.4	94.2	89.6	90.0	91.1
	InceptionResNet-V1^48^	94.1	95.4	94.6	94.3	95.5
	EfficientNet-V2^49^	97.0	95.3	92.3	96.5	96.9
	ViT^43^	93.8	93.3	93.6	94.1	93.8
Applied ML models	RF^50^	84.9	89.7	79.8	87.2	89.7
	KNN^51^	81.7	76.4	78.8	76.4	86.9
	SVM^52^	86.5	89.2	84.3	86.1	91.4
	NB^53^	88.3	92.1	86.5	84.2	92.7
**Proposed model**	**NB with GMM**	**97.6**	**99.0**	**98.1**	**97.9**	**98.5**

**Table 7:** Classification results of deep learning and traditional machine learning classifiers on the HCRF dataset. [In (%)].

**Type**	**Classifier**	**Rec.**	**Spe.**	**Pre.**	**F1**	**Acc.**
Applied DL models	Xception	96.2	96.7	94.0	96.1	96.4
	Inception-V3^45^	96.8	96.1	93.8	95.4	96.7
	AlexNet^46^	90.2	91.7	87.4	88.2	87.1
	DenseNet-121^47^	90.4	93.3	90.6	92.1	90.8
	InceptionResNet-V1^48^	96.2	90.4	92.4	93.3	94.5
	EfficientNet-V2^49^	98.1	96.3	94.3	95.5	97.1
	ViT^54^	94.8	95.2	94.1	95.0	96.3
Applied ML models	RF^50^	86.7	91.2	80.4	88.2	91.7
	KNN^51^	86.7	85.4	82.8	84.3	88.0
	SVM^52^	91.2	90.2	90.3	89.1	93.6
	NB^53^	95.3	90.2	91.5	90.8	93.3
**Proposed model**	**NB with GMM**	**98.6**	92.4	**94.4**	**96.5**	**97.3**

### Segmentation of abnormal regions

#### Qualitative results

**Fig. 5:**
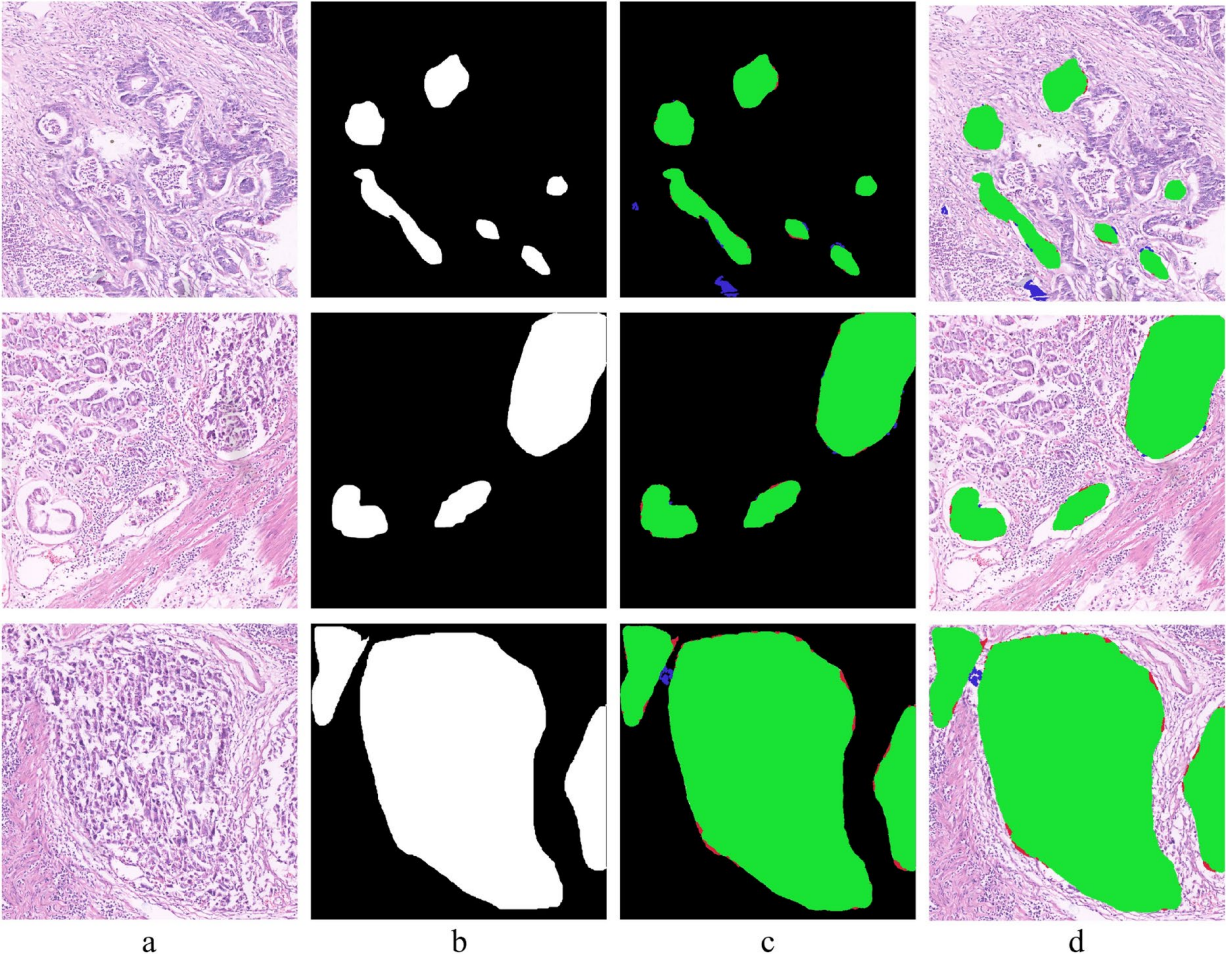
Example of whole-slide image segmentation results by the proposed model **(a)** Original H&E images with abnormal condition, **(b)** Ground truth images of the respective images in (a), **(c)** Pixel-based segmentation results of the corresponding images, where green, blue, and red color represent pixels with true positive, false positive, and false negative, **(d)** Overlay of the segmentation results in (c) on the original corresponding images in (a).

We extensively evaluated our proposed segmentation algorithm on H&E datasets to assess its ability to differentiate between abnormal regions within H&E-provided photographs. The segmentation results are illustrated in Figure 5, showing the effectiveness of the proposed methodology. Original images from the abnormal class are shown in column (a), and the corresponding GT annotations are shown in column (b). The segmentation results obtained with our suggested algorithm can be seen in column (c), and for better visual clarity, the segmentation results are superimposed on the original images in column (d).

Results of pixel-based segmentation are color-coded for easy interpretation: TPs are shown as green, FPs as blue, and FNs as red. With only a few cases of misclassification (shown in blue) and small omissions (shown in red), the segmentation in the first-row example skillfully captures GT areas (shown in green). Better segmentation outcomes are further demonstrated in the second-row example. Large aberrant regions are successfully segmented (green) in the third-row example, although there are a few minor edge oversights (red) and minimal misclassification (blue). The algorithm’s efficiency is demonstrated in the last-row example, especially in correctly segmenting relatively small abnormal regions. In conclusion, the qualitative findings support our suggested segmentation algorithm’s promising ability to distinguish abnormal areas in H&E pictures.

#### Quantitative results

An extensive evaluation of the proposed IFCM segmentation technique for identifying abnormal regions in stomach histopathology pictures may be found in the quantitative results section. Performance metrics such as recall, specificity, accuracy, precision, DC, and JI are used to assess the effectiveness of the segmentation approach. These measurements offer insight into the aberrant regions the segmentation technique identifies, which is essential for future study and diagnosis.

Recall is one indicator of the sensitivity of the segmentation method. Approximately 79% of the real aberrant regions in the histopathology images are appropriately identified by the IFCM approach. This demonstrates how numerous pathological domains emphasize its capacity to support illness detection and diagnosis. Specificity is the ability of the segmentation findings to identify the normal regions. With a specificity of 92.13%, our suggested model accurately distinguishes abnormal from normal areas. This high specificity is required to reduce FPs and improve the reliability of the segmentation findings. The proposed methodology performs strongly and achieves segmentation results similar to the GT annotations, with an accuracy of 84.68% in GHS. The precision value of 62.94% indicates that the proposed method accurately identifies problematic areas while minimizing false detection.

Furthermore, the DC and JI show the overlap and similarity between the segmented regions and the GT annotations. The segmented anomalous regions and the given GT show substantial agreement (74.21%) and moderate overlap (60.24%). These numbers show how effective the segmentation strategy is in GHS.

**Fig. 6:**
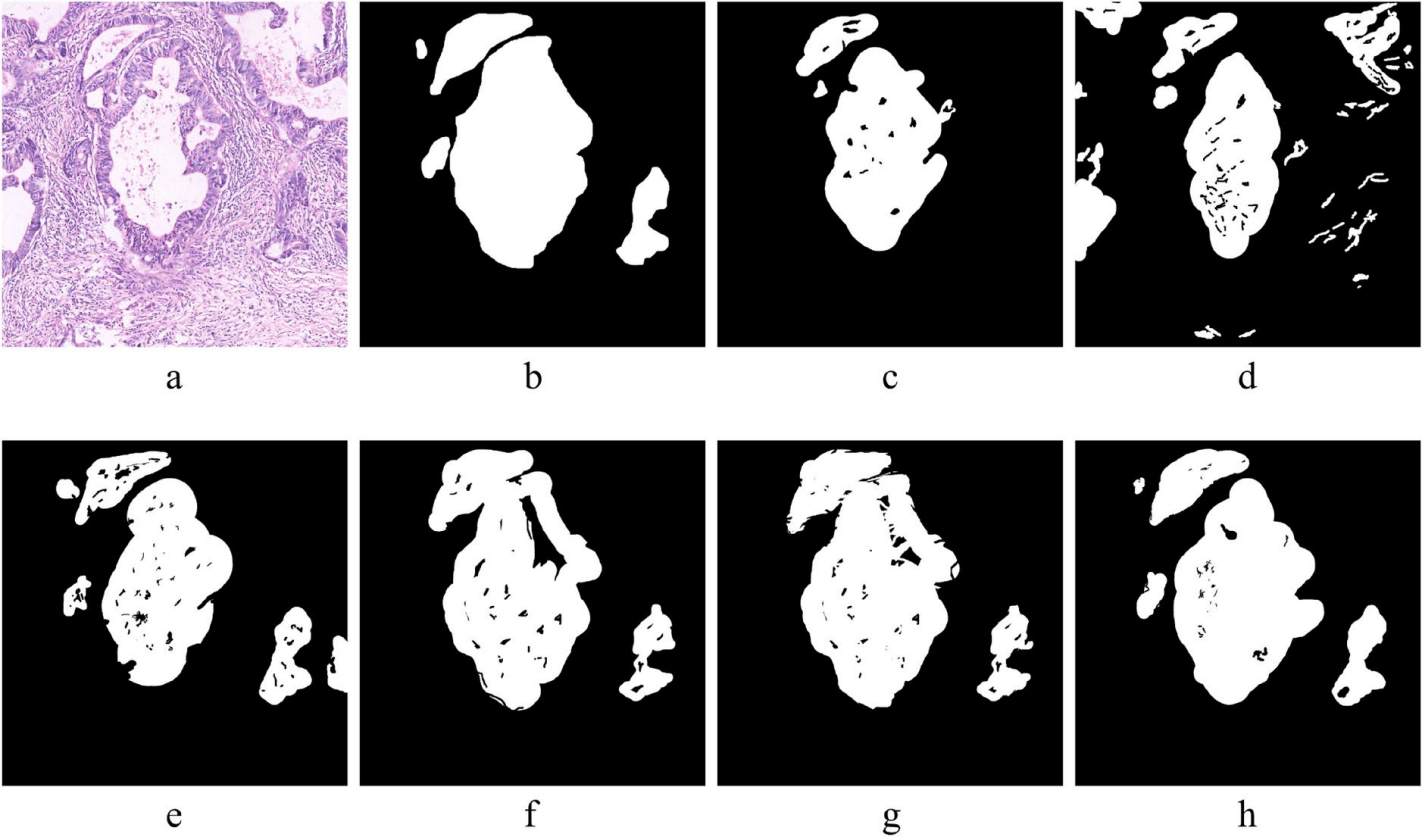
Whole-slide image segmentation result comparison of the existing techniques and the proposed model **(a)** Original histology image having abnormal region, **(b)** Ground truth image of the respective image in (a), **(c)** Segmentation results using SegNet^55^, **(d)** Watershed^56^, **(e)** U-Net^57^, **(f)** DenseCRF^58^, **(g)** CRF-RNN^59^, **(h)** Proposed technique.

Table 8 illustrates the quantitative results comparison of the existing segmentation methods with our proposed technique on the digital histopathological HCRF^41^ test dataset. The evaluation metrics highlight the superior performance of our proposed segmentation method, IFCM, in correctly identifying abnormal regions. The proposed segmentation achieved a DC and JI value of 65.21% and 60.24%, respectively. This shows that our proposed segmentation significantly improves for DC and JI values by 36.46% and 69.32%, respectively. Conclusively, the outcome results show the potential efficacy of the proposed segmentation model in detecting anomalous areas in gastric histopathology images. Reliable clinical applications are possible after looking at the GHS results for diagnostic purposes. This is evidenced by its combination of high recall, specificity, accuracy, reasonable precision, substantial agreement, and overlap with the GT annotations. We conducted a thorough comparative analysis, comparing our suggested segmentation methodology to cutting-edge methods such as SegNet^55^, Watershed^56^, U-Net^57^, DenseCRF^58^, and CRF-RNN^59^. The segmentation outcomes generated using these methods are visually illustrated in Figure 6.

**Table 8:** Performance comparison of existing segmentation methods with our proposed technique on digital histopathology images using the HCRF dataset. [In (%)].

**Approach**	**Rec.**	**Spe.**	**Acc.**	**Pre.**	**DC**	**JI**
SegNet^55^	33.25	86.12	77.01	39.87	22.14	14.28
Watershed^56^	33.17	80.22	71.55	30.35	24.13	17.67
U-Net^57^	67.96	78.58	77.82	42.14	47.57	33.91
DenseCRF^58^	70.89	79.25	79.02	42.45	47.78	34.12
CRF-RNN^59^	91.24	80.54	80.11	44.01	49.56	35.58
**IFCM**^1^	**78.84**	**92.13**	**84.68**	**62.94**	**65.21**	**60.24**

## Model’s interpretability and the role of segmentation

Segmentation of abnormal regions in histopathology gastric images plays a crucial role in both the localization and quantification of abnormality. The segmentation result allows the visualization of the model’s performance by highlighting the region of interest (ROI). The segmentation process also enables the quantification of the abnormal region. The clearly defined segmented boundaries allow the measurement of the size, shape, and area of the ROI. These metrics are essential for clinical applications, as they can provide insights vital for diagnosis, prognosis, and treatment planning. Accurate quantification of abnormal regions not only aids in assessing the severity of a condition but also in tracking its progression over time. Moreover, segmentation could also be used as a benchmark to compare different techniques. The comparison is crucial in demonstrating the overall superiority of the method, not only in classification accuracy but also in the precise identification and quantification of abnormal regions.

**Fig. 7:**
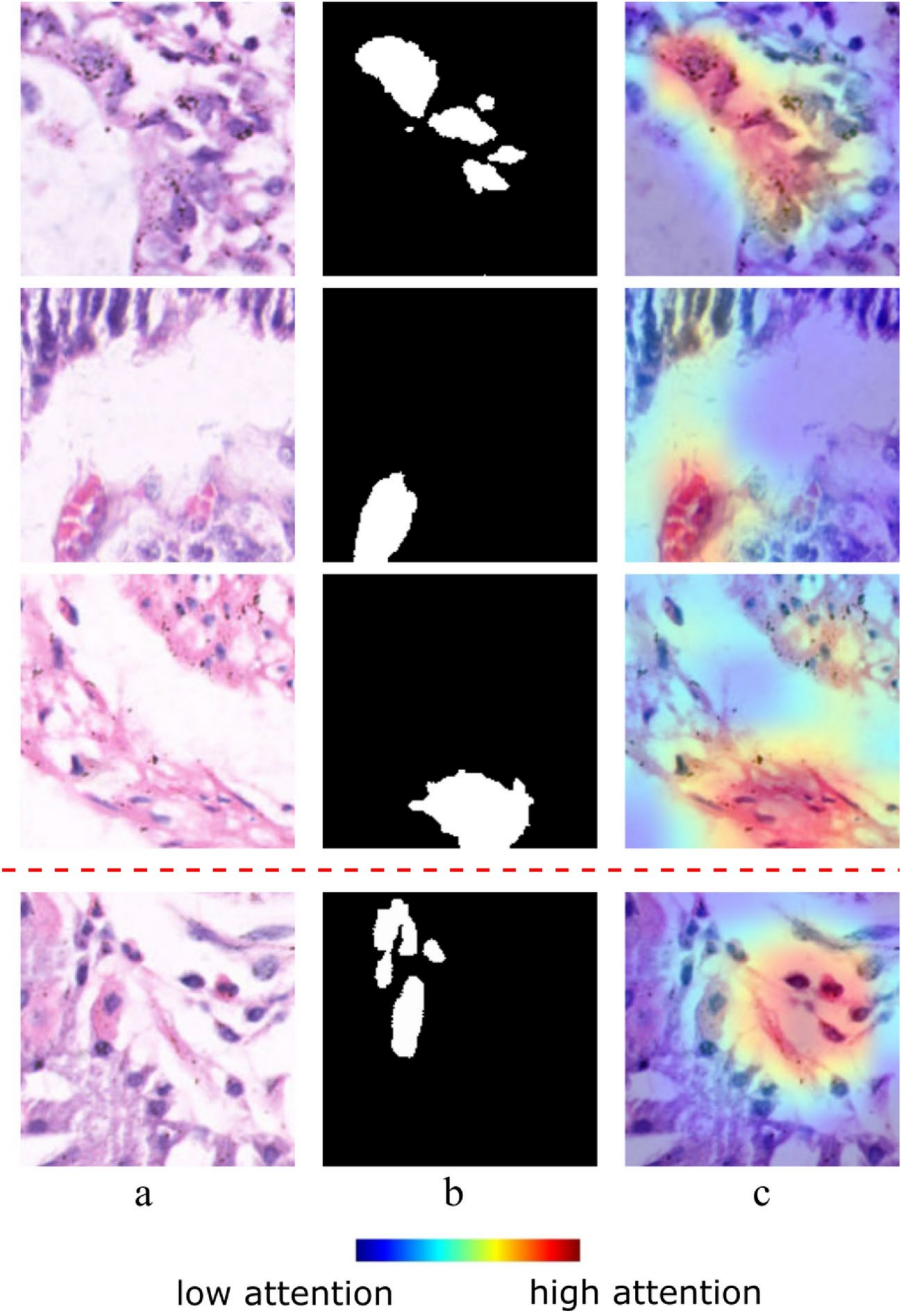
Example images with corresponding Grad-CAM heatmap showcasing correct and incorrect prediction using the proposed segmentation technique. (**a**) Original histology image with the abnormal region, (**b**) Ground truth image of the respective image, (**c**) Generated Grad-CAM with heatmap showcasing the high and low attention regions.

Interpretability is essential if an AI-driven model is to be trusted. Interpretation refers to providing a meaningful explanation for the AI model’s predictions. This involves translating the model’s internal mechanisms into human-understandable terms, i.e., through visualizations. To address the model’s interpretation, we have incorporated Grad-CAM into our proposed framework. Grad-CAM highlights the regions of the input image that the model deems most relevant to identifying abnormal regions, providing a visual explanation of the model’s decision-making process. A few examples are shown in figure 7. It is clearly visible that the highest attention and the GT areas (showing abnormality) are the same in the first three rows of the figure 7. The last row below the red dashed line represents an incorrect case where attention is not given to the GT area. The red-shaded area in the heatmap represents the region with highest attention whereas the bluish with lowest attention. The heatmap provides an informative visualization, where the Grad-CAM generated image illustrates the model’s interpretability, highlighting the areas that significantly influence the model’s decision-making process. In summary, the segmentation step significantly contributes not only to the localization and quantification of the abnormality but also to the model’s interpretation. Through Grad-CAM visualization, we provide a robust and interpretable AI-driven tool that can be trusted by clinicians for making informed decisions in gastric histopathology.

## Discussion

The discussion section of the research article highlights the significance of classification and segmentation in the context of GHCS. First, the study shows that the proposed NB classifier using GMM with EM is effective in medical image analysis, exhibiting remarkable accuracy in identifying GC. The specificity of the suggested model was slightly less than that of a DL model (Xception). It is important to remember that sensitivity matters more, particularly in screening scenarios that aim to detect all potential disease cases. Sensitivity over specificity guarantees prompt detection and treatment, reducing the chance of illness progression and enhancing patient outcomes. The possible effects of FNs on illness diagnosis make this significant. Given that the proposed classifier might be integrated into real-time screening scenarios for the early detection of GC, it has significant practical implications. The classifier facilitates early detection and intervention and expedites the screening process by swiftly and reliably categorizing histopathology images. This is especially helpful in places with little resources, where access to pricy and invasive diagnostic methods may be limited. Furthermore, the classifier’s real-time operation makes it easier to use and more accessible, making it a useful tool for clinical practice and population-wide screening initiatives. GC used to be primarily classified as binary or multi-class. By measuring the affected areas using the GC segmentation, our work goes one step further. When evaluating the extent and trajectory of the illness, experts can greatly benefit from the segmentation in their research.

This study proposed an improved segmentation strategy by making it possible to identify aberrant regions in histopathology images with higher accuracy. Comparing the improved results to other segmentation methods shows how precisely it can detect and quantify the abnormality-affected areas. This work represents a major advancement in the diagnostic potential of medical imaging, allowing for more accurate and reliable assessments of GC. Clinicians can design more efficient treatment plans and attain superior therapeutic results by precisely recognizing anomalies in space. Additionally, this would provide prompt interventions and treatment plan updates by tracking the anomaly’s spread. The produced results offer perceptions on how to enhance the efficacy and precision of image-based diagnostic techniques. The suggested NB classifier’s results indicate that GC classifiers employing histopathological images can succeed. The innovative algorithmic methods make it a potentially helpful tool in the screening of GC. The future refinement of the classifier may enhance its clinical utility and facilitate a more comprehensive integration within healthcare environments.

## Data Availability

The datasets used in this study are publicly accessible^40,41^. To access, you can download them directly from the following websites: https://gitee.com/neuhwm/GasHisSDB (GasHisSDB), and https://data.mendeley.com/datasets/thgf23xgy7/2 (HCRF).

## Code availability

The code is publicly available by accessing the following link:https://github.com/zubairfarooqi/GHCS/.

## References

[1] Wild, C. P., Stewart, B. W. & Wild, C. *World cancer report 2014* (World Health Organization Geneva, Switzerland, 2014).

[2] Garcia, E. et al. Automatic lymphocyte detection on gastric cancer ihc images using deep learning. In 2017 IEEE 30th international symposium on computer-based medical systems (CBMS), 200–204 (IEEE, 2017).

[3] Wang, F.-H. *et al.* The chinese society of clinical oncology (csco): clinical guidelines for the diagnosis and treatment of gastric cancer. *Cancer communications*** 39**, 1–31 (2019).30606259

[4] Hohenberger, W., Weber, K., Matzel, K. & Papadopoulos, T. Standardized surgery for gastric cancer-german version. *Oncology Research and Treatment*** 43**, 689–696 (2020).

[5] Lozano, R. Comparison of computer-assisted and manual screening of cervical cytology. *Gynecologic oncology ***104**, 134–138 (2007). 16959306 10.1016/j.ygyno.2006.07.025

[6] Sies, K. *et al.* Past and present of computer-assisted dermoscopic diagnosis: performance of a conventional image analyser versus a convolutional neural network in a prospective data set of 1,981 skin lesions. *European Journal of Cancer*** 135**, 39–46 (2020). 32534243 10.1016/j.ejca.2020.04.043

[7] Bi, D., Zhu, D., Sheykhahmad, F. R. & Qiao, M. Computer-aided skin cancer diagnosis based on a new meta-heuristic algorithm combined with support vector method. *Biomedical Signal Processing and Control ***68**, 102631 (2021).

[8] Malibari, A. A. *et al.* Optimal deep neural network-driven computer aided diagnosis model for skin cancer. *Computers and Electrical Engineering ***103**, 108318 (2022).

[9] Abràmoff, M. D. *et al.* Automated and computer-assisted detection, classification, and diagnosis of diabetic retinopathy. *Telemedicine and e-Health*** 26**, 544–550 (2020). 32209008 10.1089/tmj.2020.0008 PMC7187982

[10] Haider, A. *et al.* Artificial intelligence-based computer-aided diagnosis of glaucoma using retinal fundus images. *Expert Systems with Applications ***207**, 117968 (2022).

[11] Zubair, M. *et al.* A comprehensive computer-aided system for an early-stage diagnosis and classification of diabetic macular edema. *Journal of King Saud University-Computer and Information Sciences*** 35**, 101719 (2023).

[12] Zubair, M. et al. Automated grading of diabetic macular edema using color retinal photographs. In 2022 2nd International Conference of Smart Systems and Emerging Technologies (SMARTTECH), 1–6 (IEEE, 2022).

[13] Zubair, M., Ali, H. & Javed, M. Y. Automated segmentation of hard exudates using dynamic thresholding to detect diabetic retinopathy in retinal photographs. *J. Multim. Process. Technol.*** 7**, 109–116 (2016).

[14] Zubair, M., Yamin, A. & Khan, S. A. Automated detection of optic disc for the analysis of retina using color fundus image. In 2013 IEEE International Conference on Imaging Systems and Techniques (IST), 239–242 (IEEE, 2013).

[15] Zubair, M., Khan, S. A. & Yasin, U. U. Classification of diabetic macular edema and its stages using color fundus image. *Journal of Electronic Science and Technology*** 12**, 187–190 (2014).

[16] Mahmood, T., Arsalan, M., Owais, M., Lee, M. B. & Park, K. R. Artificial intelligence-based mitosis detection in breast cancer histopathology images using faster r-cnn and deep cnns. *Journal of clinical medicine ***9**, 749 (2020). 32164298 10.3390/jcm9030749 PMC7141212

[17] Aljuaid, H., Alturki, N., Alsubaie, N., Cavallaro, L. & Liotta, A. Computer-aided diagnosis for breast cancer classification using deep neural networks and transfer learning. *Computer Methods and Programs in Biomedicine ***223**, 106951 (2022). 35767911 10.1016/j.cmpb.2022.106951

[18] Owais, M. *et al.* Multilevel deep-aggregated boosted network to recognize covid-19 infection from large-scale heterogeneous radiographic data. *IEEE Journal of Biomedical and Health Informatics*** 25**, 1881–1891 (2021). 33835928 10.1109/JBHI.2021.3072076 PMC8545161

[19] Khan, S. H. *et al.* Covid-19 detection and analysis from lung ct images using novel channel boosted cnns. *Expert Systems with Applications ***229**, 120477 (2023). 37220492 10.1016/j.eswa.2023.120477 PMC10186852

[20] Zubair, M., Umair, M. & Owais, M. Automated brain tumor detection using soft computing-based segmentation technique. In 2023 3rd International Conference on Computing and Information Technology (ICCIT), 211–215 (IEEE, 2023).

[21] Woźniak, M., Siłka, J. & Wieczorek, M. Deep neural network correlation learning mechanism for ct brain tumor detection. *Neural Computing and Applications*** 35**, 14611–14626 (2023).

[22] Chen, B., Zhang, L., Chen, H., Liang, K. & Chen, X. A novel extended kalman filter with support vector machine based method for the automatic diagnosis and segmentation of brain tumors. *Computer Methods and Programs in Biomedicine*** 200**, 105797 (2021). 33317871 10.1016/j.cmpb.2020.105797

[23] Doi, K. Computer-aided diagnosis in medical imaging: historical review, current status and future potential. *Computerized medical imaging and graphics ***31**, 198–211 (2007). 17349778 10.1016/j.compmedimag.2007.02.002 PMC1955762

[24] An, P. *et al.* A deep learning method for delineating early gastric cancer resection margin under chromoendoscopy and white light endoscopy. *Gastric Cancer*** 23**, 884–892 (2020). 32356118 10.1007/s10120-020-01071-7

[25] Ling, T. *et al.* A deep learning-based system for identifying differentiation status and delineating the margins of early gastric cancer in magnifying narrow-band imaging endoscopy. *Endoscopy ***53**, 469–477 (2021). 32725617 10.1055/a-1229-0920

[26] Zhang, K. *et al.* Early gastric cancer detection and lesion segmentation based on deep learning and gastroscopic images. *Scientific Reports*** 14**, 7847 (2024). 38570595 10.1038/s41598-024-58361-8 PMC10991264

[27] Takemoto, S. *et al.* Computer-aided demarcation of early gastric cancer: a pilot comparative study with endoscopists. *Journal of Gastroenterology*** 58**, 741–750 (2023). 37256409 10.1007/s00535-023-02001-x

[28] Yan, T. *et al.* Semantic segmentation of gastric polyps in endoscopic images based on convolutional neural networks and an integrated evaluation approach. *Bioengineering*** 10**, 806 (2023). 37508833 10.3390/bioengineering10070806 PMC10376250

[29] Wang, X. *et al.* Predicting gastric cancer outcome from resected lymph node histopathology images using deep learning. *Nature communications*** 12**, 1637 (2021). 33712598 10.1038/s41467-021-21674-7 PMC7954798

[30] Sun, M. *et al.* Accurate gastric cancer segmentation in digital pathology images using deformable convolution and multi-scale embedding networks. *IEEE access ***7**, 75530–75541 (2019).

[31] Liang, Q. *et al.* Weakly supervised biomedical image segmentation by reiterative learning. *IEEE Journal of biomedical and health informatics*** 23**, 1205–1214 (2018). 29994489 10.1109/JBHI.2018.2850040

[32] Han, C. *et al.* Multi-layer pseudo-supervision for histopathology tissue semantic segmentation using patch-level classification labels. *Medical Image Analysis*** 80**, 102487 (2022). 35671591 10.1016/j.media.2022.102487

[33] Sun, C. *et al.* Gastric histopathology image segmentation using a hierarchical conditional random field. *Biocybernetics and Biomedical Engineering*** 40**, 1535–1555 (2020).

[34] Rai, H. M. Cancer detection and segmentation using machine learning and deep learning techniques: A review. *Multimedia Tools and Applications*** 83**, 27001–27035 (2024).

[35] Ali, S. *et al.* Assessing generalisability of deep learning-based polyp detection and segmentation methods through a computer vision challenge. *Scientific Reports ***14**, 2032 (2024). 38263232 10.1038/s41598-024-52063-x PMC10805888

[36] Atmakuru, A. et al. Deep learning in radiology for lung cancer diagnostics: A systematic review of classification, segmentation, and predictive modeling techniques. Expert Systems with Applications 124665 (2024).

[37] Oyelade, O. N., Ezugwu, A. E., Venter, H. S., Mirjalili, S. & Gandomi, A. H. Abnormality classification and localization using dual-branch whole-region-based cnn model with histopathological images. *Computers in Biology and Medicine*** 149**, 105943 (2022). 35986967 10.1016/j.compbiomed.2022.105943

[38] Liu, X., Jiao, L., Li, L., Tang, X. & Guo, Y. Deep multi-level fusion network for multi-source image pixel-wise classification. *Knowledge-Based Systems*** 221**, 106921 (2021).

[39] Ahmed, H., Le, C. P. & La, H. M. Pixel-level classification for bridge deck rebar detection and localization using multi-stage deep encoder-decoder network. *Developments in the Built Environment ***14**, 100132 (2023).

[40] Hu, W. *et al.* Gashissdb: A new gastric histopathology image dataset for computer aided diagnosis of gastric cancer. *Computers in biology and medicine ***142**, 105207 (2022). 35016101 10.1016/j.compbiomed.2021.105207

[41] Sun, C., Li, C. & Li, Y. Data for hcrf. Mendeley Data, v2, http://dx. doi. org/10.17632/thgf23xgy7 **2** (2020).

[42] Reinhard, E., Adhikhmin, M., Gooch, B. & Shirley, P. Color transfer between images. *IEEE Computer graphics and applications*** 21**, 34–41 (2001).

[43] Hu, W. *et al.* A comparative study of gastric histopathology sub-size image classification: From linear regression to visual transformer. *Frontiers in Medicine ***9**, 1072109 (2022). 36569152 10.3389/fmed.2022.1072109 PMC9767945

[44] Chollet, F. Xception: Deep learning with depthwise separable convolutions. In Proceedings of the IEEE conference on computer vision and pattern recognition, 1251–1258 (2017).

[45] Szegedy, C., Vanhoucke, V., Ioffe, S., Shlens, J. & Wojna, Z. Rethinking the inception architecture for computer vision. In Proceedings of the IEEE conference on computer vision and pattern recognition, 2818–2826 (2016).

[46] Krizhevsky, A., Sutskever, I. & Hinton, G. E. Imagenet classification with deep convolutional neural networks. Advances in neural information processing systems **25** (2012).

[47] Howard, A. et al. Searching for mobilenetv3. In Proceedings of the IEEE/CVF international conference on computer vision, 1314–1324 (2019).

[48] Szegedy, C., Ioffe, S., Vanhoucke, V. & Alemi, A. Inception-v4, inception-resnet and the impact of residual connections on learning. In Proceedings of the AAAI conference on artificial intelligence, vol. 31 (2017).

[49] Tan, M. & Le, Q. Efficientnetv2: Smaller models and faster training. In International conference on machine learning, 10096–10106 (PMLR, 2021).

[50] Breiman, L. *Random forests. Machine learning ***45**, 5–32 (2001).

[51] Guo, G., Wang, H., Bell, D., Bi, Y. & Greer, K. Knn model-based approach in classification. In On The Move to Meaningful Internet Systems 2003: CoopIS, DOA, and ODBASE: OTM Confederated International Conferences, CoopIS, DOA, and ODBASE 2003, Catania, Sicily, Italy, November 3-7, 2003. Proceedings, 986–996 (Springer, 2003).

[52] Cortes, C. & Vapnik, V. *Support-vector networks. Machine learning*** 20**, 273–297 (1995).

[53] Yang, F.-J. An implementation of naive bayes classifier. In 2018 International conference on computational science and computational intelligence (CSCI), 301–306 (IEEE, 2018).

[54] Dosovitskiy, A. et al. An image is worth 16x16 words: Transformers for image recognition at scale. arXiv preprint arXiv:2010.11929 (2020).

[55] Badrinarayanan, V., Kendall, A. & Cipolla, R. Segnet: A deep convolutional encoder-decoder architecture for image segmentation. *IEEE transactions on pattern analysis and machine intelligence ***39**, 2481–2495 (2017). 28060704 10.1109/TPAMI.2016.2644615

[56] Huang, Y.-L. & Chen, D.-R. Watershed segmentation for breast tumor in 2-d sonography. *Ultrasound in medicine & biology*** 30**, 625–632 (2004). 15183228 10.1016/j.ultrasmedbio.2003.12.001

[57] Siddique, N., Paheding, S., Elkin, C. P. & Devabhaktuni, V. U-net and its variants for medical image segmentation: A review of theory and applications. *Ieee Access*** 9**, 82031–82057 (2021).

[58] You, M., Luo, C., Zhou, H. & Zhu, S. Dynamic dense crf inference for video segmentation and semantic slam. *Pattern Recognition ***133**, 109023 (2023).

[59] Xu, X., Zhou, F. & Liu, B. Automatic bladder segmentation from ct images using deep cnn and 3d fully connected crf-rnn. *International journal of computer assisted radiology and surgery ***13**, 967–975 (2018). 29556905 10.1007/s11548-018-1733-7

